# Characterizing the Tumor Immune Microenvironment with Tyramide-Based Multiplex Immunofluorescence

**DOI:** 10.1007/s10911-021-09479-2

**Published:** 2021-02-15

**Authors:** Hidetoshi Mori, Jennifer Bolen, Louis Schuetter, Pierre Massion, Clifford C. Hoyt, Scott VandenBerg, Laura Esserman, Alexander D. Borowsky, Michael J. Campbell

**Affiliations:** 1 Center for Immunology and Infectious Diseases, University of California, Davis, CA USA; 2 Department of Pathology, University of California San Francisco, San Francisco, CA USA; 3 Department of Medicine, Vanderbilt University Medical Center, Nashville, TN USA; 4 Akoya Biosciences Inc, Marlborough, MA USA; 5 Department of Surgery, University of California San Francisco, San Francisco, CA USA; 6 Mt Zion Carol Franc Buck Breast Care Center, University of California San Francisco, San Francisco, CA USA; 7 Department of Pathology and Laboratory Medicine, School of Medicine, University of California Davis, Sacramento, CA USA

**Keywords:** Breast cancer, Immune cells, Immunohistochemistry, Multiplex

## Abstract

Multiplex immunofluorescence (mIF) allows simultaneous antibody-based detection of multiple markers with a nuclear counterstain on a single tissue section. Recent studies have demonstrated that mIF is becoming an important tool for immune profiling the tumor microenvironment, further advancing our understanding of the interplay between cancer and the immune system, and identifying predictive biomarkers of response to immunotherapy. Expediting mIF discoveries is leading to improved diagnostic panels, whereas it is important that mIF protocols be standardized to facilitate their transition into clinical use. Manual processing of sections for mIF is time consuming and a potential source of variability across numerous samples. To increase reproducibility and throughput we demonstrate the use of an automated slide stainer for mIF incorporating tyramide signal amplification (TSA). We describe two panels aimed at characterizing the tumor immune microenvironment. Panel 1 included CD3, CD20, CD117, FOXP3, Ki67, pancytokeratins (CK), and DAPI, and Panel 2 included CD3, CD8, CD68, PD-1, PD-L1, CK, and DAPI. Primary antibodies were first tested by standard immunohistochemistry and single-plex IF, then multiplex panels were developed and images were obtained using a Vectra 3.0 multispectral imaging system. Various methods for image analysis (identifying cell types, determining cell densities, characterizing cell-cell associations) are outlined. These mIF protocols will be invaluable tools for immune profiling the tumor microenvironment.

## Introduction

The interface of cancer with the host immune system occurs primarily in the reactive tissue stroma around the tumor and this has been referred to as the tumor immune microenvironment (TIME). The TIME has come under renewed and intense interest with the success of cancer immunotherapy using immune checkpoint inhibitors that target proteins such as programmed cell death protein 1 (PD-1, a T-cell co-inhibitory receptor) or programmed death ligand 1 (PD-L1, also called B7-H1 or CD274) [1, 2]. Characterizing the TIME in the context of immunotherapy can help generate candidate predictive biomarkers of response. In addition, since various immune cell populations are involved in both pro- and anti-cancer responses, a better understanding of these cells and their associations with each other and with the cancer cells will help guide the identification of new immunotherapeutic strategies.

Immunohistochemistry is routinely used for pathological analysis of important clinical markers such as estrogen receptor or HER2. However, standard IHC protocols typically only measure a single marker per slide. With the recent advent of multiplex immunofluorescence (mIF), this limitation is overcome by allowing detection of multiple different markers on a single tissue section [3–6]. One strategy for mIF utilizes sequential rounds of antibody-labelling of one marker, followed by horseradish peroxidase (HRP) catalyzed linking of fluorophore-conjugated tyramide molecules around the antibody-labelled epitope. The fluorophore is covalently bound to tyrosine residues on or around the marker of interest, allowing for the primary and secondary antibodies to be stripped from the section before the next round of staining. The mIF stained slides can then be scanned with a multispectral imaging microscope and analyzed with a variety of image analysis software packages.

For mIF to be widely adopted as a diagnostic and prognostic tool, staining and imaging protocols need to be standardized, automated, and validated. Automated slide stainers are relatively common equipment in clinical laboratories. Here we present an optimized protocol for fully-automated seven-color mIF incorporating TSA coupled with a multispectral imaging system for the simultaneous detection of six tissue biomarkers plus a nuclear counterstain. We developed two panels which included the following markers: CD3, CD8, CD20, CD68, CD117, FOXP3, PD-1, PD-L1, Ki67, pancytokeratins (CK), and DAPI. The pancytokeratins and Ki67 markers allowed analysis of epithelial tumor cell boundaries and the proliferation status of various cell types, respectively. In addition, we present image analysis methods for phenotyping cells, determining cell densities, and characterizing cell-cell associations.

## Materials and Methods

### Tissue Specimens

All tissues used in this study were provided without patient identification or clinical information. Some were obtained in the context of other clinical studies with appropriate institution review board oversight and informed consent for those studies, but these data were not shared with our research team. As such, this work was determined to be “not human subject research” under the Public Health Service definition. Sequential 4 µm sections from human tonsil FFPE blocks were prepared for conventional immunohistochemistry (IHC), single-plex immunofluorescence, and multiplex immunofluorescence (mIF) staining. In addition, 4 µm sections from cases of lung carcinoma and breast cancer were prepared for mIF.

### Single-plex IHC and IF

The antibody clones used in this study are shown in Table 1. Chromogenic staining (DAB single-plex) was performed on the Ventana Discovery Ultra autostainer (Roche). 4 µm tonsil FFPE sections were mounted on Superfrost Plus microscope slides (Thermo Fisher Scientific) and dried overnight prior to staining. Sections were deparaffinized followed by antigen retrieval in CC1 buffer (pH 9, 95 °C; Roche), endogenous peroxidase blocking, and then incubation with the primary antibodies. Chromogenic detection was performed with Chromomap DAB (Roche) followed by counterstaining with hematoxylin. Tonsil sections stained with and without primary antibody were used as positive and negative controls for each marker. Working dilutions were determined on the basis of the negative and positive stained sections as well as the expected staining pattern of cells for the given marker.

**Table1.** Staining conditions for multiplex IHC for IP1 and IP2

Staining cycle	Marker	Clone	Company	Product	Antibody dilution	Fluorophore	Fluorophore dilution
IP1							
1	FOXP3	SP97	Spring	M3972	1:25	Opal620	1:250
2	CKs	AE1/AE3	DAKO	M3515	1:200	Opal650	1:200
3	Ki67	30-9	Ventana	790-4286	RTU	Opal690	1:100
4	CD20	L26	Ventana	790-2531	RTU	Opal540	1:250
5	CD3	2GV6	Ventana	790-4341	RTU	Opal520	1:100
6	CD117	c-kit	DAKO	A4502	1:100	Opal570	1:300
7			Perkin Elmer	FP1490		DAPI	RTU
IP2							
1	PDL1	E1L3N	CST	13648e	1:100	Opal620	1:100
2	PD1	EPR4877	Abcam	ab137132	1:100	Opal650	1:200
3	CD8	4B11	Leica	CD8-4B11-L-CE	1:100	Opal690	1:100
4	CKs	AE1/AE3	DAKO	M3515	1:200	Opal570	1:300
5	CD68	PG-M1	DAKO	M0876	1:100	Opal540	1:250
6	CD3	2GV6	Ventana	790-4341	RTU	Opal520	1:100
7			Perkin Elmer	FP1490		DAPI	RTU

After chromogenic IHC, single-plex IF was performed for each marker. We first assigned Opal fluorophores (Akoya Biosciences) to each marker and tested the primary antibody at its optimal dilution determined from the chromogenic staining, as well as one dilution above and one dilution below this. Staining was performed on the Ventana Discovery Ultra. Sections were deparaffinized followed by antigen retrieval in CC1 at 97 °C. To inhibit enzymatic activities, Discovery inhibitor (Roche) was applied, followed by the primary antibody. Detection was performed with OmniMap secondary antibody (Roche) followed by incubation with one of the Opal fluorophores, prepared according to the manufacturer’s instructions. Finally, the slides were counterstained with Discovery QD DAPI (Roche) or Spectral DAPI (Akoya Biosciences) and mounted with ProLong Diamond antifade mounting medium (Thermo Fisher Scientific). As with the IHC single-plex staining, positive and negative controls were included and the optimal titration in the single-plex IF was chosen based on a uniform, specific, and correct staining pattern.

### Automated mIF Staining

A schematic of the mIF staining workflow is shown in Supplementary Figure 1. After each primary antibody was optimized in single-plex IHC and IF, they were combined to create two 6-marker multiplex panels. To test each panel, tonsil sections were stained with primary antibodies at the optimized concentrations determined from the single-plex assays. Sections were subjected to 6 sequential rounds of staining with each primary antibody followed by a secondary HRP-conjugated polymer. Signal amplification was achieved with TSA-Opal fluorophores. Between each round of staining, a heat-induced epitope retrieval (HIER) step was performed to remove primary-secondary-HRP complexes before staining with the next primary antibody. After the final round of antibody staining, slides were counterstained with Discovery QD DAPI or Spectral DAPI and mounted with ProLong Diamond antifade mounting medium.

**Fig. 1:**
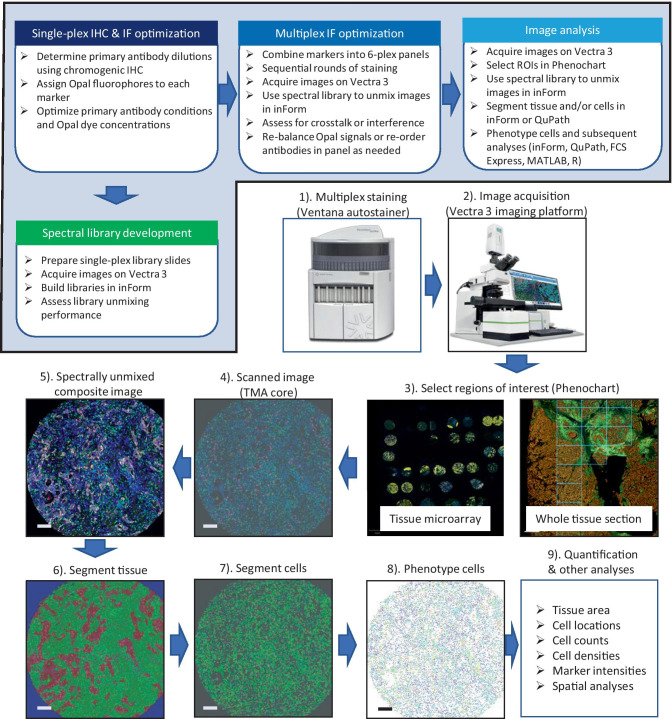
Workflow for multiplex immunofluorescence panel optimization and image analysis. The top flow diagram highlights the mIF panel optimization steps, from single-plex IHC & IF optimization to spectral library development, multiplex IF optimization, and image analysis. The lower flow diagram highlights the steps used for image acquisition and analysis. (**1**) Whole slides containing tumor tissue or tissue microarrays were stained on a Ventana Discovery Ultra platform. (**2**) Stained slides were scanned with the Vectra 3 at low magnification (4x or 10x). (**3**) Regions of interest were selected in Phenochart on tissue microarray (TMA) or whole tissue section. (**4**) High-resolution acquisition (20x) of ROIs. (**5**) Images obtained were imported into inForm and spectrally unmixed using a previously optimized spectral library. (**6**) Using inForm’s (or QuPath) trainable tissue segmentation algorithms, images were segmented into tumor, stroma, and no tissue regions. (**7**) Using inForm (or QuPath), nuclei were identified and cell segmentation was performed. (**8**) Cells were phenotyped based on the labeling of markers in each mIF panel. (**9**) Data of individual cells derived from phenotyping in inForm, QuPath, or FCS Express were exported for further analyses. Scale bar on each TMA core is 100 µm

During multiplex optimization, antibody-Opal dye pairings, concentrations, and order in the panel were assessed and adjusted as needed. This was achieved by checking signal-to-noise ratios (signal intensity of positive stain:background > 10:1) and signal balance (signal intensity of each fluorophore ~10-30 normalized counts) using inForm software (Akoya Biosciences). To check for signal crosstalk, a leave-one-out set of controls was run for each antibody using the final optimized mIF protocol. For each panel, six serial sections from human tonsil tissue were prepared. Each section was subjected to the full mIF protocol with one primary antibody left out. These were imaged on the Vectra 3, then the images were unmixed and analyzed using inForm. The final sequence of antibodies for each panel is shown in Table 1, along with the antibody/Opal dye pairs and their working dilutions. Full protocols for mIF staining (panels IP1 and IP2) on the Ventana Discovery autostainer are presented in Supplementary Tables 1 and 2.

### Manual mIF Staining

Tissue sections (4 µm) prepared from formalin-fixed paraffin embedded tissue blocks were baked at 58 °C for 1 hour. If tissue detachment (commonly exhibited as lifting edges of sections) was observed following a staining procedure, a longer baking time was applied to revised protocols to enhance tissue attachment. After baking, tissue sections were deparaffinized with three treatments of xylene for 5 min each, followed by three rinses with 100% ethanol for 2 min each. Sections were rehydrated by rinsing in 95% ethanol, 70% ethanol, and water for 5 min each. Initial antigen retrieval was performed in a decloaking chamber (Biocare Medical LLC, Concord, CA) with 10mM citrate buffer (pH 6.0) for 45 min at 125 °C at 15psi.

An Opal 7-color manual IHC kit (Akoya Bioscience) was used for manual mIF staining with the same antibodies and Opal dye dilutions used for automated staining (Table 1). Tissue sections were incubated with the first primary antibody diluted in Antibody Diluent/Blocking solution provided in the kit followed by washing in TBST buffer and then incubated with the secondary-HRP conjugate. After another wash, slides were incubated with the appropriate Opal fluorophore. The HIER step for removing the primary-secondary-HRP complex between staining rounds was achieved by placing the slides in a plastic coplin jar filled with antigen retrieval buffer (AR6 provided in the kit), placing the jar in a 1100-watt microwave, and heating for 45 min at 100% power followed by 14 min at 20% power. After the slides cooled to room temperature they were washed and then were ready for the next round of staining with the next primary antibody. After the last round of antibody staining, slides were counterstained with Spectra DAPI, then mounted with Fluoromount-G Mounting Medium (Thermo Fisher Scientific).

### Image Acquisition and Analysis

Stained slides were imaged using the Vectra 3 quantitative pathology imaging system (Akoya Biosciences). A tonsil section was included as a positive control in each batch of mIF stained tumor sections run on the autostainer. After a low magnification scan (4x or 10x), regions of interest (ROI) were selected using the Phenochart viewer (Akoya Bioscience) and these ROIs were subsequently scanned/acquired at a higher resolution (20x). For tissue microarrays (TMAs), each core section was considered an ROI. To build a spectral library for subsequent spectral unmixing of these images, single-plex IF stains were performed on tonsil sections with an anti-CD20 primary antibody paired with each of the Opal fluorophores (without a DAPI counterstain). An unstained tonsil section and a DAPI only stained section were also prepared. Representative fields from these single-color slides were imaged at 20x using the Vectra 3, and spectra were extracted from the acquired images using inForm software and saved to a spectral library. The quality of the spectral library was assessed by evaluating unmixed images to confirm the absence of spectral overlap or bleed-over between channels.

Image files acquired on the Vectra 3 were opened in inForm and spectrally unmixed using the appropriate spectral library. Several image analysis software packages were then used to identify and characterize cells in the ROIs. These included inForm, QuPath, FCS Express, MATLAB, and various R packages. The use of these platforms will be described in more detail in the Results section.

## Results

### Multiplex Immunofluorescence Panel Optimization

We designed two 6-plex mIF panels based on recommendations of the Molecular and Cellular Characterization of Screen-Detected Lesions (MCL: https://mcl.nci.nih.gov/) pathologists working group. The first panel, designated IP1, consisted of the markers CD3, CD20, FOXP3, CD117, pan-cytokeratins (CK), and Ki67. The second panel, IP2, was comprised of the markers CD3, CD8, CD68, PD-1, PD-L1, and CK. A schematic depicting the workflow for developing and optimizing these panels is shown in Fig. 1. Evaluation/optimization of single-plex staining is fundamental in designing a multiplex protocol as it determines the staining parameters for each individual marker in the mIF panel. Initial working dilutions for single-plex IF were determined on the basis of single-plex IHC stained sections that yielded the expected staining pattern of cells for each marker. Initial antibody-Opal dye pairings were assigned to each marker based on the single-plex IHC results, taking into consideration the expression and relative abundance of each marker.

**Fig. 2:**
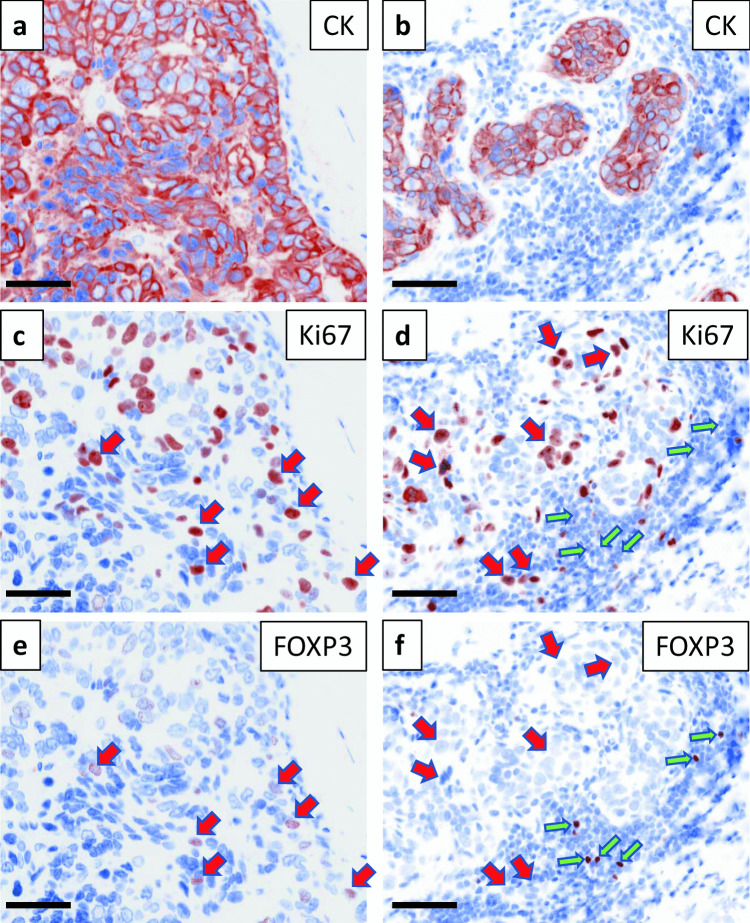
Spectral bleed through in IP1 resolved by changing antibody-Opal dye pairings. Panels** a**, **c**, and **e** show staining of a breast cancer section with IP1 in which Ki67 was paired with Opal 570 and FOXP3 was paired with Opal 620. As indicated by the red arrows, most of the Ki67 positive cells were showing up as FOXP3 positive cells, indicating spectral bleed over or incomplete stripping of the Ki67 antibody. Panels** b**, **d**, and **f** show the results of a redesigned IP1 in which Ki67 swapped positions and Opal pairing with CD117 yielding Ki67/Opal 690 and CD117/Opal 570 (with FOXP3 remaining paired with Opal 620). The red arrows highlight some cells that are Ki67^+^ and FOXP3^-^ and the green arrows highlight some cells that are FOXP3^+^ and Ki67^-^. Scale bars are 50 µm

Once the optimal staining parameters were established for each marker, the single-plex IF protocols were combined into a preliminary mIF protocol. Initial staining sequences for each mIF panel were arranged such that sequential antibodies do not colocalize in the same cellular compartments within the same cells. This helps when issues of incomplete stripping arise. In addition, since Opal 520 and Opal 570 show some attenuation by HIER, these fluorophores were placed in later rounds of staining. The multiplex protocols were progressively modified as needed for reasons such as spectral bleed through or inadequate antibody stripping. This included revising antibody-Opal dye pairings, positions, and dilutions. Following each adjustment to the protocol, the signal-to-background ratio and signal balance for each marker were reassessed. As an example, the initial design of IP1 paired Ki67 with Opal 570 and FOXP3 with Opal 620. However, as shown in Fig. 2, Ki67 positive tumor cells were appearing as FOXP3 positive cells, visualized here using inForm’s pathology view option (panels A, C, and E). This suggested either bleed through of the Opal 570 signal into the Opal 620 channel, or incomplete stripping of the Ki67 antibody (which was last in the sequence). Redesigning this mIF panel to pair Ki67 with Opal 690 and CD117 with Opal 570 (leaving FOXP3 paired with Opal 620) resolved this issue (Fig. 2, panels b, d, and f). This put the Ki67/Opal690 in a channel further away from the FOXP3/Opal620 pairing and in addition, placed Ki67 3^rd^ in the staining sequence, which was then followed by more HIER stripping steps compared to the initial placement of Ki67 in the last position of the staining sequence.

**Fig. 3:**
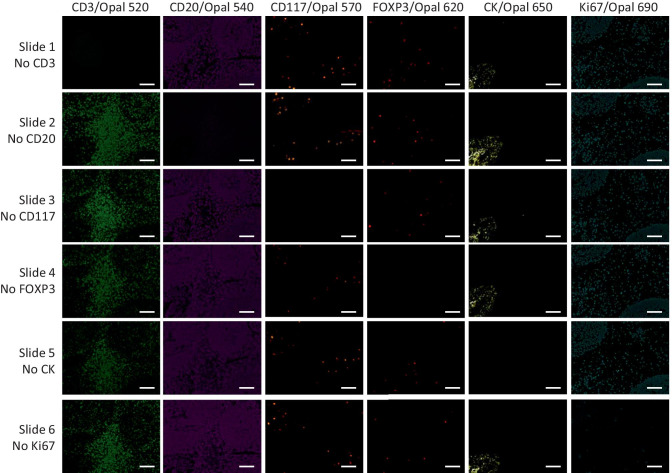
Leave-one-out control stains for IP1. Six serial sections from human tonsil FFPE tissue were prepared and stained with the full IP1 protocol. Each row represents images from a slide in which one primary antibody was left out of the staining protocol (ex. Row 1, Slide 1, no CD3 had anti-CD3 left out). Each image in a row shows the staining pattern for the antibody/fluorophore combination shown at the top, with all other channels turned off. The absence of spectral bleed through or inadequate antibody stripping issues are indicated by the lack of signal in the images along the diagonal (top left to bottom right). Scale bars are 100 µm

Leave-one-out control stains were performed for the final optimized protocols for mIF panels IP1 and IP2 to evaluate cross-talk issues (Fig. 3 and Supplementary Figure 2). Each row of images in Fig. 3 is from a tonsil section stained with IP1 with one of the primary antibodies left out of the protocol. Each image in a row shows the signal from the individual channel for the antibody-Opal dye pairs shown across the top of the figure. For example, the top row, Slide 1, had anti-CD3 left out, so there is no signal in the CD3/Opal 520 channel, but there are signals in all of the other channels. Similarly, Slide 2 had anti-CD20 left out, and here we see signal in all images except for the one corresponding to the CD20/Opal 540 channel. The lack of signals in the images along the diagonal (top left to bottom right) confirmed the absence of spectral bleed through or inadequate antibody stripping issues. Similar results were obtained for IP2 (Supplementary Figure 2).

### Image Acquisition and Region of Interest Selection for mIF Analysis

After mIF panels were optimized, they were applied to lung cancer and breast cancer tissue samples. Using the Vectra 3 imaging system, a low magnification whole slide scan (4x or 10x) was initially obtained. For large tissue sections, it is often necessary to confine the image analysis to several regions of interest as opposed to the entire section. These can be selected using the Phenochart viewer and are subsequently scanned at a higher resolution (20x) (see Fig. 1). There are various ROI selection strategies including random selection of ROIs, ROI selection based on staining hot spots, or ROI selection based on other criteria (eg. tumor/stroma borders). However, there are no established guidelines for ROI selection, so we recommend that the selection methodology be described when presenting results from mIF analyses.

**Fig. 4:**
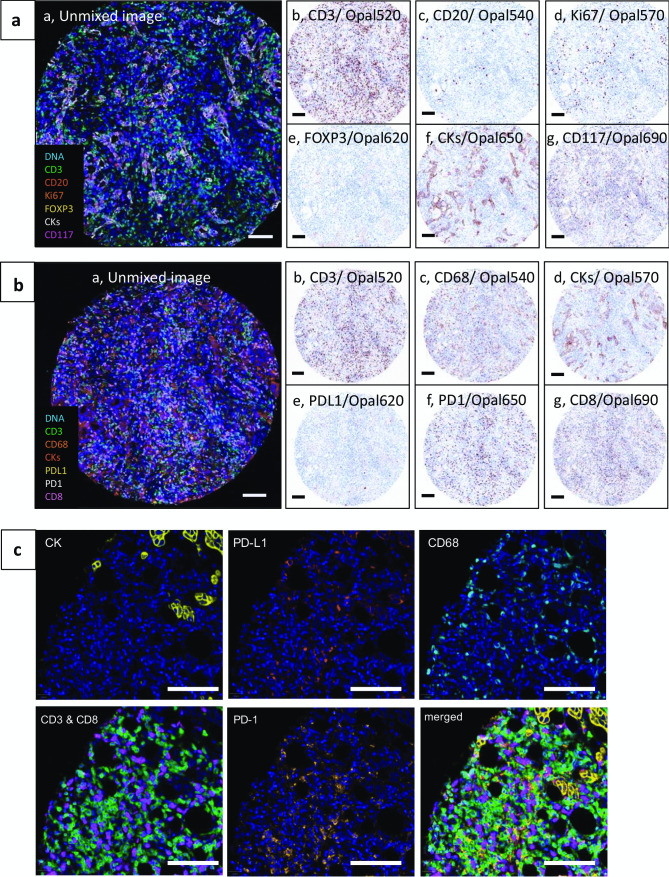
Representative examples of mIF with IP1 and IP2 on tumor tissue. A human bronchial airway biopsy TMA was stained with mIF panels IP1 **A** and IP2 **B**. Composite images from a representative core, pseudo-colored as indicated in the legends, are shown in panels Aa and Ba. Pathology views from inForm (positive signal = brown, DAPI counterstain = blue) for each individual marker are shown in panels **b**-**g**. Scale bars are 100 µm. **C** A human breast cancer tissue stained with IP2 illustrating the co-localization of PD-1^+^ T cells with PD-L1^+^ macrophages. Each image is of the same region of the tissue with only the indicated marker’s channels turned on. Merged: CK (yellow), CD3 (green), CD8 (magenta), CD68 (cyan), PD-1 (orange), PD-L1 (red), DAPI (blue). Scale bars are 100 µm

For tissue microarrays (TMAs), each core is typically defined as an ROI (Fig. 1). As an example, images of a bronchial airway tumor TMA stained with optimized mIF protocols for IP1 and IP2 are shown in Fig. 4. After staining, slides were scanned on the Vectra 3 with each core treated as an ROI in Phenochart. Multispectral images for each core were opened in inForm and spectrally unmixed. Unmixed composite images of a representative core stained with IP1 (Fig. 4a panel a) and IP2 (Fig. 4b panel a) are shown with each marker pseudo-colored as indicated in the legends. “Pathology views” for each individual marker are also shown. These were generated in inForm, where the specific marker/Opal dye is pseudo-colored brown to mimic a DAB chromogenic stain and the DAPI channel is colored blue to mimic a hematoxylin counterstain. IP1 identifies 5 different cell types in the tumor microenvironment: CD3^+^ T cells, CD20^+^ B cells, CD3^+^FOXP3^+^ regulatory T cells (Treg), CD117^+^CK^-^ mast cells, and CK^+^ epithelial/tumor cells (Fig. 4a panels b-g). The co-localization of nuclear Ki67 staining within these cell types permits the evaluation of their proliferation status. In addition, co-localization of CD117 with CK can distinguish three populations of cells: CK^+^CD117^+^ tumor cells, CK^+^CD117^-^ tumor cells, and CK^-^CD117^+^ mast cells. IP2 identifies 4 different cell types: CD3^+^CD8^-^ T cells, CD3^+^CD8^+^ cytotoxic T cells, CD68^+^ macrophages, and CK^+^ epithelial/tumor cells (Fig. 4b panels b-g). This panel also includes the immune checkpoint molecules PD-1 and PD-L1 whose expression in any of these cell types can be evaluated. As an example, Fig. 4c shows a region from a breast cancer TMA stained with IP2 demonstrating co-localization of PD-L1^+^CD68^+^ cells with PD-1^+^ T cells (both CD3^+^CD8^-^ and CD3^+^CD8^+^).

### Image Analysis Using inForm Software

After ROIs have been selected, scanned, and spectrally umixed in inForm, a variety of imaging platforms can be used to analyze the images. Unmixed multispectral component images can be analyzed within inForm itself, or these images can be exported from inForm and opened with other software packages such as QuPath [7], ImageJ [8–10], and HALO (Indica Labs).

The first step in image analysis is often segmentation of the tissue into different categories such as tumor, stroma, or no tissue (Fig. 1). inForm contains a user-trainable protocol to automatically detect and segment specific tissue types based on tissue morphologies using machine-learning feature recognition algorithms. Training is performed by drawing a training area that encompasses the tissue category of interest (eg. stroma, tumor, or no tissue). Specific markers can be used to help identify different tissue compartments, for example CK staining to differentiate epithelial tumor cells from stromal cells. Segmentation is iteratively refined by adding new training areas to address any misclassified regions. Training a tissue segmentation algorithm is typically performed on several images and then the final trained algorithm can be applied to a batch of similarly stained images.

**Fig. 5:**
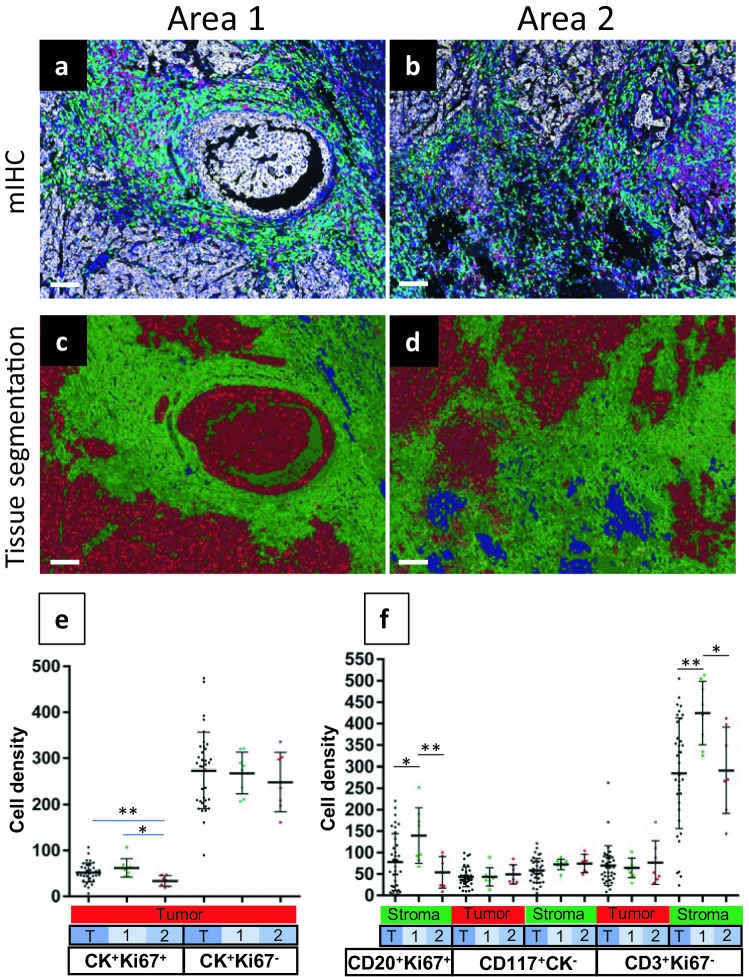
A comparison of areas from a breast cancer tissue demonstrating differences in densities of immune cells. Panels** a **&** b**: Unmixed composite images from two ROIs selected from two different areas of a breast cancer tissue (see Supplementary Figure 3) stained with IP1 (CK/white, CD3/green, CD20/yellow, CD117/red and DAPI/blue are indicated). Panels** c **&** d**: Tissue segmentation of a and b showing tumor (red), stroma (green), and no tissue (blue) areas. Scale bars are 100 µm. (**e**) Cell densities of proliferating tumor cells (CK^+^Ki67^+^) and non-proliferating tumor cells (CK^+^Ki67^-^) within the tumor segmented regions across the entire tissue (T) or just within area 1 or area 2 as indicated. (**f**) Cell densities of various immune cell populations within the tumor or stroma segmented regions across the entire tissue (T) or just within area 1 or area 2 as indicated. For both Panels **e** and **f**, each dot represents the cell density within one ROI. Horizontal lines indicate mean values. Student’s T test: *p<0.05, **p<0.01

Figure 5 illustrates the tissue segmentation algorithm applied to a breast cancer sample. After staining with IP1, two areas of interest containing both tumor and stroma were selected for analysis (Supplementary Figure 3). Unmixed composite images from two ROIs selected from these areas are shown in Fig. 5, panels a and b. CK^+^ tumor cells are pseudocolored white in these images. Panels c and d show the tissue types identified by a trained tissue segmentation algorithm where red areas indicate tumor, green areas indicate stroma, and blue areas indicate no tissue present. A comparison of Fig. 5a to 5c and Fig. 5b to 5d illustrates the robustness of the tissue segmentation algorithm.

Following tissue segmentation, cell segmentation and phenotyping was performed. Using inForm, nuclei were identified/segmented using the DAPI counterstain and a variety of user-defined parameters such as DAPI intensity, minimum nuclear size, and splitting sensitivity. Following nuclear segmentation, parameters for membrane/cytoplasm segmentation were adjusted, including cytoplasm thickness to define the distance from the boundary of each cell’s nucleus to the outer boundary of its cytoplasm and membrane search distance, which defines the maximum distance from the boundary of each cell’s nucleus to search for membrane signals.

Once cell segmentation parameters were adequately optimized, cell phenotyping was performed. inForm’s phenotyping feature automatically classifies cell phenotypes using machine-learning algorithms to differentiate cell types across a tissue section. Phenotypes for IP1 and IP2 were defined based on the markers present in the panel, along with a phenotype designated as “other” for cells that weren’t positive for any of the markers. For training the classifier, the software requires only 5 user selected cells as examples for each marker. However, we have found that generally, 20-30 examples of each marker are required for reliable cell phenotyping. In addition, example cells should be selected across several images. Training typically involved selecting an initial set of ~10 example cells for each marker, followed by iterative addition of examples to refine the classifier. When the training is complete, the algorithm can be applied to a batch of similarly stained sections. Due to staining variability across tissue samples, the phenotyping results must be verified for each case and a new classifier may need to be trained for individual cases. Finally, the data obtained following phenotyping were exported from inForm and tabulated reports of cell numbers, percentages, or cell densities (counts per area) were obtained using the R package phenoptr [11] or phenoptr Reports (Akoya Biosciences). The phenoptr package contains functions that make it easy to read and analyze data tables and images created in inForm.

Figure 5 (E&F) illustrates an inForm analysis applied to a breast cancer sample stained with IP1 (Supplementary Figure 3). Thirty-six ROIs, including both tumor and stroma, were selected across the entire tissue section. Two areas were selected to investigate whether sampling smaller regions adequately represents the overall tumor tissue, with respect to immune cell infiltrates (Supplementary Figure 3). Eight ROI images were obtained from area 1 and seven from area 2. Tissue segmentation, cell segmentation, and phenotype analyses using inForm were performed on the 36 ROIs obtained from this breast cancer section. Phenoptr was used to generate cell density data (cell counts per megapixel) from the inForm results. As shown in Fig. 5e, there was no difference in the density of CK^+^Ki67^-^ cells obtained for the whole tumor section compared to the two smaller sub-sampled regions. In contrast, the density of proliferating CK^+^ cells (CK^+^Ki67^+^) was significantly lower in area 2 compared to area 1 or the total tissue.

We also compared immune cell densities across the two sub-sampled regions with the total tumor section. These included B cell (CD20^+^Ki67^-^ and CD20^+^Ki67^+^), mast cell (CD117^+^CK^-^), T cell (CD3^+^Ki67^-^ and CD3^+^Ki67^+^), and regulatory T cell (CD3^+^FOXP3^+^) populations. Most immune cell populations showed similar cell densities across the different areas. However, as shown in Fig. 5f, the density of proliferating B cells (CD20^+^Ki67^+^) was significantly higher in the stroma of area 1 compared to area 2 or the total area. Likewise, non-proliferating T cells (CD3^+^Ki67^-^) were significantly higher in the stroma of area 1. These observations illustrate the spatial heterogeneity with the tumor microenvironment, with respect to immune cell infiltrates as well as proliferation status of tumor cells.

### Image Analysis Using QuPath

Unmixed multispectral component images generated in inForm are saved as multilayer TIFF files. These image files can be opened in a variety of image analysis software packages, including QuPath [7]. QuPath is an open-source software for bioimage analysis that is used for digital pathology applications due to its powerful set of tools for working with whole slide images. Like inForm, QuPath contains user-trainable algorithms for tissue segmentation, nuclear/cell segmentation, and cell phenotyping which involve iterative training/testing to optimize the classifiers.

**Fig. 6:**
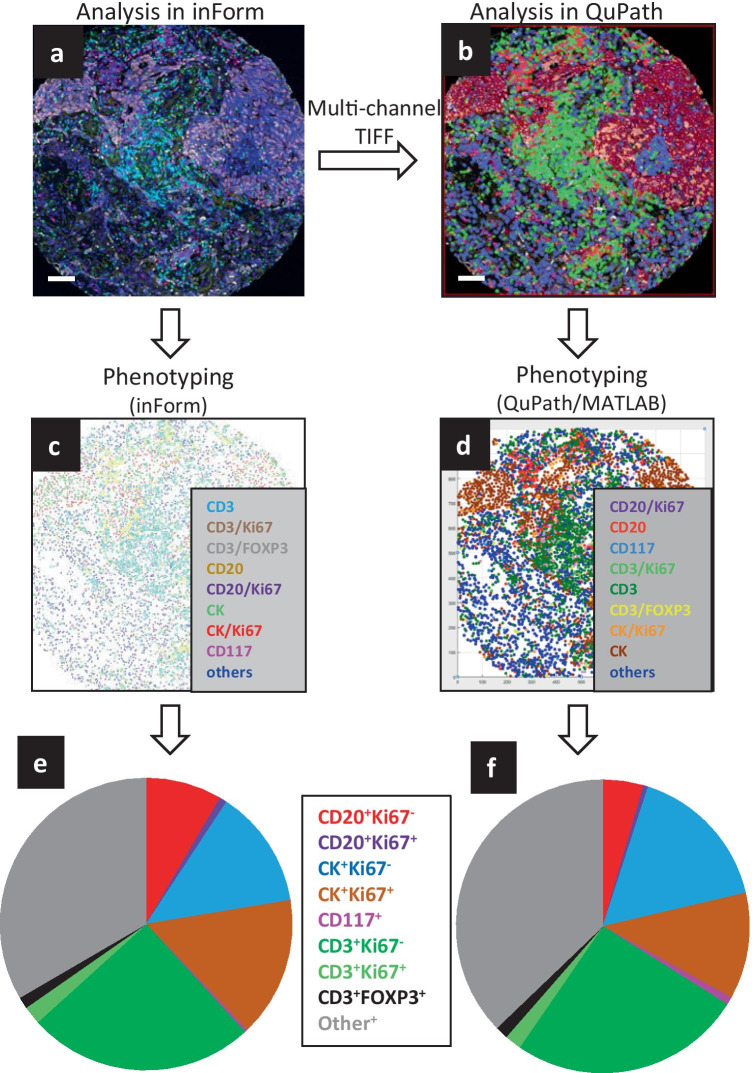
Cell phenotyping using inForm vs QuPath. Multispectral images from a TMA core stained with IP1 were unmixed in inForm (**a**) and converted to a multilayered TIFF file that was analyzed in QuPath (**b**). Scale bars are 100 µm. (**c**) Phenotype map generated in inForm. (**d**)Phenotype map created in MATLAB using phenotypes generated in QuPath. (**e**) Pie chart showing the distribution of various cell types as determined from inForm phenotyping. (**f**) Pie chart showing the distribution of various cell types as determined from QuPath phenotyping

We compared the analysis of a TMA core image in inForm with analysis of the same image in QuPath. Figure 6 shows the pseudo-colored, unmixed composite image obtained from inForm (panel a) and the pseudo-colored image obtained from the component TIFF file imported into QuPath (panel b). A phenotype map obtained after segmentation and phenotyping in inForm is shown in Fig. 6c and a similar map, plotted in MATLAB after segmentation and phenotyping in QuPath, is shown in Fig. 6d. Cell densities obtained from both platforms are shown in the pie charts (Fig. 6e and 6f) and demonstrate that both analysis pipelines yielded similar results, with the exception of a higher fraction of CD20^+^Ki67^-^ B cells being identified in this image by the inForm analysis.

### Image Analysis Using FCS Express

Image analysis software such as inForm and QuPath provides sufficient information on a per cell basis, including total fluorescence for each fluorophore and cell size, to be processed using flow cytometry software. FCS Express 6 Image Cytometry software (DeNovo Software) can import mIF images and associated data that were acquired using the Vectra imaging platform. This allows users to apply flow cytometry gating strategies to digital microscopy images with access to the original images for visualization.

**Fig. 7:**
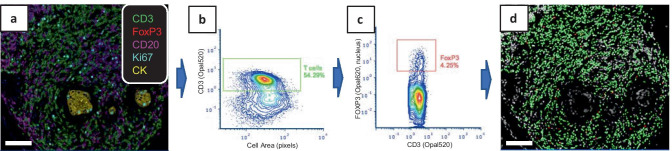
Cell phenotyping using gating strategies in FCS Express. (**a**) Unmixed composite image of a breast cancer tissue stained with IP1. (**b**) Contour plot of cell size vs. CD3 mean fluorescence generated from inForm data that were imported into FCS Express. A T cell gate (green rectangle) was manually drawn around the CD3^+^ cell population. (**c**) CD3 vs. FOXP3 contour plot of gated T cells from b. A Treg gate (red) was manually drawn around the FOXP3^+^ cell population. (**d**) Masks of gated cells (T cells in green, Treg cells in red) overlaid on a grayscale image of the nuclear DAPI channel. Scale bars are 100 µm

In Fig. 7, we show an example of gating Treg cells in a breast cancer sample using FCS Express. The tissue was stained with IP1, images were acquired on the Vectra system, and cell segmentation was performed in inForm. The composite image (TIFF file; Fig. 7a), component images (multi-image TIFF), segmentation maps (multi-image TIFF), and cell segmentation data files were exported from inForm and imported into FCS Express. A contour plot of cell size vs. CD3 fluorescence was generated in FCS Express and a rectangular gate was drawn around the population of CD3^+^ cells as shown in Fig. 7b. Cells in this gate were then used to create a plot of CD3 vs. nuclear FOXP3 staining for gating Treg cells (CD3^+^FOXP3^+^; Fig. 7c). As gates are being drawn, one can visualize in real time which cells are being included in the gates as shown in Fig. 7d (CD3 gated cells are marked in green and Tregs are marked in red). The ability to inspect the data at the level of the nucleus (as with the FOXP3 staining shown in Fig. 7), cytoplasm, membrane or total fluorescence provides a benefit over standard flow cytometry analyses which generally measure total cellular fluorescence and require additional staining protocols to obtain data on subcellular location. Cell counts, percentages, and other statistics can be generated from the gated populations in FCS Express, and exported as reports or as data tables for further analyses.

FCS Express also includes several other data visualization tools (histograms, dot plots, density plots), reporting tools (bar charts, pie charts, line graphs), and high-dimensional data reduction tools such as tSNE, SPADE and K-means, which can be applied to multispectral data generated from mIF. Finally, since flow cytometry is commonly used in research and clinical settings, this type of analysis is easily understood by immunology researchers and clinicians who are familiar with results from flow cytometry software, providing image data in a manner that is readily transferable between research and diagnostic laboratories.

### Additional Analyses of Cell Phenotype Data

**Fig. 8:**
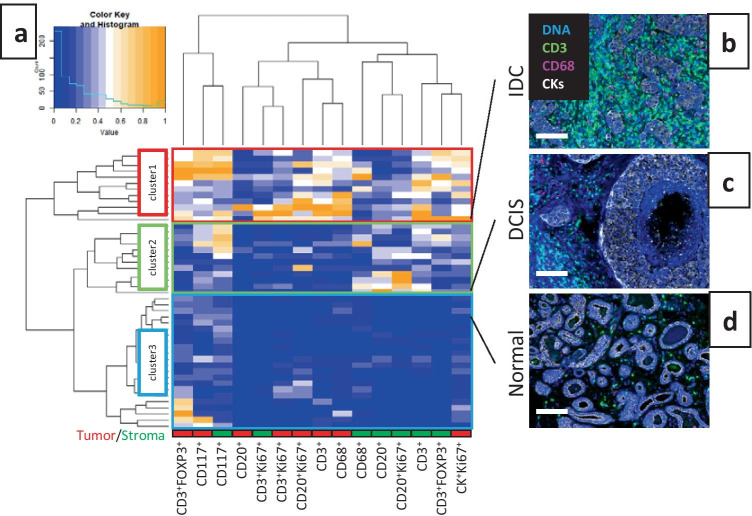
Heatmap clustering of immune cells in different regions of a breast cancer sample. A breast cancer tissue sample was stained with mIF panels IP1 and IP2 and analyzed using inForm. Selection of ROIs from this sample is shown in Supplementary Figure 4. The heatmap panel **a** shows the relative densities of various cell populations, where each row represents data from one ROI and each column is a cell population within the tumor or stroma compartments as indicated along the bottom. Unsupervised clustering yielded 3 main clusters as indicated on the left. ROIs were annotated as invasive ductal carcinoma (IDC), ductal carcinoma in situ (DCIS), or normal. A representative ROI composite image from each of the different annotations and their corresponding row in the heatmap is shown in panels **b**, **c**, and **d**. CD3 (green), CD68 (magenta), CK (white), and DAPI (blue) are highlighted

After cells have been segmented and phenotyped, for example using inForm, QuPath, or FCS Express, the data can be exported for further analyses in other software packages. R provides a number of data analysis and visualization packages. One of these, phenotpr, was designed specifically for the analysis of data generated from inForm [11]. As an example of using R packages to analyze mIF image data, we compared immune cells infiltrates in various regions of a breast cancer tissue section using a clustered heatmap analysis. Sections were stained with IP1 and IP2 and 46 ROIs from each were acquired on the Vectra system (Supplementary Figure 4). These ROIs were annotated by a pathologist to be representative of either IDC, DCIS, or normal tissue. Cell segmentation and phenotyping were performed in inForm and the resulting data were exported for subsequent analysis. Cell densities were determined for each ROI using phenoptr and a clustered heatmap was generated with the heatmap.2 function in the R package gplots [12]. This heatmap (Fig. 8) illustrates the relative densities of various cell types within the different areas of the tumor. Unsupervised cluster analysis yielded 3 main clusters, denoted cluster 1, 2, and 3 in Fig. 8. Cluster 1 had high levels of immune infiltrates, cluster 2 had moderate levels, and cluster 3 had low levels. In addition, cluster 1 had higher levels of proliferating CK^+^ cells (CK^+^Ki67^+^) compared to the other clusters. All of the ROIs annotated as normal tissue fell into cluster 3. Of the DCIS annotated ROIs, 52.2% belonged to cluster 3, 43.5% to cluster 2, and only 4.3% to cluster 1. Finally, 90% of the ROIs annotated as IDC fell into cluster 1, 10% in cluster 2, and 0% in cluster 3. This clustering heatmap analysis provides a convenient method to visualize and compare the differences in ROIs across a tissue sample.

**Fig. 9:**
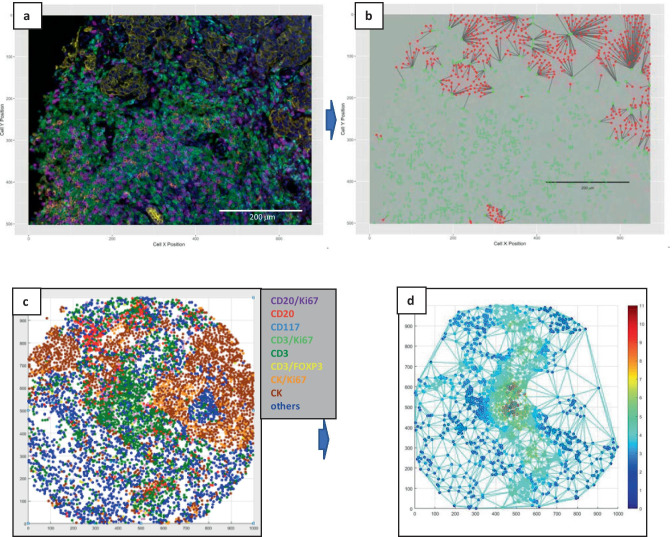
Spatial profiling of cells within the tumor microenvironment. (a) Unmixed composite image of a breast cancer sample stained with mIF panel IP2 (CK/yellow, CD3/green, CD8/magenta, CD68/cyan, PD-1/orange, PD-L1/red, DAPI/blue). **b** Nearest neighbor analysis of **a** using phenoptr R package. Line segments are drawn from each tumor cell (red dots) to its nearest CD8^+^ T cell (green dots). Scale bars are 200 µm for (a) and (b). **c** Phenotype map created in MATLAB from IP1 staining of a TMA phenotyped in QuPath. **d** Delaunay triangulation neighborhood plot for CD3^+^ T cells from **c** (each dot represents a T cell) overlaid with a heatmap representing the density of Treg cells within 100 µm of each T cell

The analyses in Fig. 5 and 8 illustrates the spatial heterogeneity of immune infiltrates across different areas within a breast cancer specimen. Since the spatial location (x,y coordinates) for each cell in an image can be obtained from inForm or QuPath analyses, these data along with the phenotyping data can be used to evaluate spatial associations between various cell types in the tumor microenvironment. Nearest neighbor analysis quantifies cell-cell interactions by determining the distance between each cell of one phenotype and its closest neighbor of another phenotype. The phenoptr package includes functions for calculating nearest neighbor distances, counting cells within a radius of a given cell type, and plotting/visualizing nearest neighbors. Figure 9 shows a composite image of a breast cancer sample stained with IP2 (Fig. 9a) and a nearest neighbor map (from phenoptr) depicting CK^+^ tumor cells in red and CD8^+^ T cells in green, with a line drawn from every tumor cell to the nearest CD8^+^ T cell (Fig. 9b). Similar analyses can be performed in MATLAB. Here we show a phenotype map generated in MATLAB from cell segmentation and phenotyping data obtained in QuPath (Fig. 9c). Fig. 9d depicts a Delaunay triangulation neighborhood plot for the CD3^+^ T cells in this sample (each dot represents a T cell). The density of Treg cells within 100 µm of each T cell is displayed as a heatmap above each dot and shows a high clustering of Treg and T cells near the center of the image.

## Discussion

There is a large clinical need to characterize immune cell infiltrates in cancers. Immune biomarkers will be important for stratifying patients to various immunotherapies as well as determining why some patients do not respond to or relapse following current immunotherapy. This will require complex phenotyping using a variety of markers and is currently beyond the scope of standard chromogenic IHC that is widely used in the clinic. There is a sea of information hiding in a tissue section that is only partially revealed by standard IHC and this is where multiplex immunofluorescence (mIF) platforms will become indispensable tools for elucidating the interplay between the immune system and cancer.

The widespread adoption of mIF techniques for diagnostic pathology will require optimized, validated automation protocols. Manual mIF staining is time consuming and the multiple rounds of staining/stripping potentially introduces unacceptable levels of variability across time and between individuals performing the staining. Herein, we described the development and optimization of protocols for two 7-color TSA-based mIF panels using Opal fluorophores on a Ventana Discovery Ultra autostainer. For both panels, single-plex IHC and IF stains were initially evaluated. The optimization of mIF protocols involved fine-tuning primary antibody and fluorophore dilutions and incubation times, testing different epitope retrieval buffers, identifying optimal HIER incubation periods and temperatures, testing different sequences of primary antibodies, and testing different pairings of primary antibodies with Opal fluorophores. Spectral crosstalk or incomplete stripping of antibodies between rounds of staining was ruled out using leave-one-out controls. In our initial optimization of IP1, leave-one-out controls revealed some bleed-through of Ki67 stain into the FOXP3 channel. This was remedied by changing the antibody-Opal dye pairings.

In addition to optimizing the staining protocol, the use of multispectral imaging aids in the identification of positively stained cells. Multispectral imaging and unmixing reduces fluorophore crosstalk and increases the signal-to-noise ratio by capturing the spectral signal of each fluorophore while isolating tissue autofluorescence. The Vectra 3 provides multispectral and automated imaging within a familiar digital pathology workflow. This workflow can include the Phenochart whole slide viewer with annotation capability, where users (pathologists, technicians, etc.) can navigate around a whole slide scanned image and identify areas of interest for high-resolution multispectral acquisition.

A variety of imaging platforms can be used to analyze scanned and spectrally unmixed images including inForm, QuPath, ImageJ, FCS Express, and HALO. These platforms utilize different approaches for the identification and phenotyping of cells, from user-trainable machine-learning algorithms (inForm, QuPath) to manual or semi-automated gating strategies (FCS Express). Therefore, the analysis and interpretation of mIF data needs to be carefully planned and should involve the participation of a multidisciplinary team, including technicians, immunologists, pathologists, and biostatisticians.

One of the main advantages of this platform when compared to other recently developed approaches such as single-cell RNA sequencing and CyTOF [13, 14], is the preservation of tissue architecture. Characterizing the spatial proximity of different cell types within the tumor microenvironment may uncover novel biomarkers of response. For example, is it more important to have a high density of T cells or a high fraction of tumor cells with at least one T cell within close proximity? Or, is the proximity of effector T cells to Treg cells more informative than the Treg:T cell ratio? To address these types of questions requires the preservation of tissue architecture. There are other mIF platforms that offer much larger plex panels than the method used herein while still preserving tissue architecture, such as CODEX (Akoya Biosciences) [15], Digital Spatial Profiling (Nanostring)[16, 17], InSituPlex (Ultivue)[18], and mass spectrometry based platforms [19, 20]. However, these have many limitations that do not permit cost- and time-effective whole-slide imaging.

To conclude, we have presented a digital pathology workflow for the optimization and automation of mIF panels for use in cancer immunology studies. We have described the development of two mIF panels, IP1 and IP2, designed to characterize immune infiltrates in cancer. IP1 includes markers for T cells (CD3), Treg cells (FOXP3), B cells (CD20), mast cells (CD117), tumor/epithelial cells (CK), and a proliferation marker (Ki67). IP2 includes markers for T cells (CD3), cytotoxic T cells (CD8), macrophages (CD68), tumor/epithelial cells (CK), and two immune checkpoint molecules (PD-1 and PD-L1). We are currently working on expanding this repertoire of immune markers and developing panels that will include other immune inhibitory checkpoints (such as CTLA4, TIM-3, LAG-3, TIGIT, VISTA), immune stimulatory checkpoints (such as OX40, GITR, CD40, CD137, ICOS, CD27), innate immunity checkpoints (CD47), NK cell inhibitory receptors (KIRs, CD96), as well as panels to better elucidate myeloid lineage cells (macrophages, dendritic cells, myeloid derived suppressor cells) in the tumor microenvironment.

## Supplementary Information

Below is the link to the electronic supplementary material.

Supplementary file1: Figure 1. Schematic of the multiplex immunofluorescence staining workflow. Figure 2. Leave-one-out control stains for IP2. Six serial sections from human tonsil FFPE tissue were prepared and stained with the full IP2 protocol. Each row represents images from a slide in which one primary antibody was left out of the staining protocol. Each image in a row shows the staining pattern for the antibody/fluorophore combination shown at the top, with all other channels turned off. The absence of spectral bleed through or inadequate antibody stripping issues are indicated by the lack of signal in the images along the diagonal (top left to bottom right). Scale bars are 100 µm. Figure 3. Example of a breast cancer tissue stained with mIF panel IP1. Phenochart image of a breast cancer tissue stained with IP1. Red boxes indicate Areas 1 and 2 that were selected for the analysis shown in Figure 5. Green boxes indicate the two ROIs shown in Figure 5A&C and 5B&D, respectively. Scale bar is 1 mm. Figure 4. Example of a breast cancer tissue stained with mIF panels IP1 and IP2. Phenochart images of serial sections of a breast cancer tissue stained with IP1 (A) and IP2 (B) are shown. Boxes indicate ROIs selected for the analysis shown in Figure 8. Scale bars are 4 mm. (PDF 1092 KB)

Supplementary file2: Table 1. IP1 protocol summary for Ventana Discovery. Table 2. IP2 protocol summary for Ventana Discovery. (PDF 548 KB)

## Data Availability

It can be supplied if necessary.

## Code Availability

Software used for acquiring dataset (inForm) and MATLAB are licensed software. QuPath and R(RStudio) are open-source software.
