# Supplementary material for: Characterizing the Tumor Immune Microenvironment with Tyramide-Based Multiplex Immunofluorescence
Source: J Mammary Gland Biol Neoplasia. 2021 Feb 15;25(4):417–32. doi: 10.1007/s10911-021-09479-2 (PMC7960613; doi:10.1007/s10911-021-09479-2)
Supplement: Supplementary file 1 — Supplementary file1: Figure 1. Schematic of the multiplex immunofluorescence staining workflow. Figure 2. Leave-one-out control stains for IP2. Six serial sections from human tonsil FFPE tissue were prepared and stained with the full IP2 protocol. Each row represents images from a slide in which one primary antibody was left out of the staining protocol. Each image in a row shows the staining pattern for the antibody/fluorophore combination shown at the top, with all other channels turned off. The absence of spectral bleed through or inadequate antibody stripping issues are indicated by the lack of signal in the images along the diagonal (top left to bottom right). Scale bars are 100 µm. Figure 3. Example of a breast cancer tissue stained with mIF panel IP1. Phenochart image of a breast cancer tissue stained with IP1. Red boxes indicate Areas 1 and 2 that were selected for the analysis shown in Figure 5. Green boxes indicate the two ROIs shown in Figure 5A&C and 5B&D, respectively. Scale bar is 1 mm. Figure 4. Example of a breast cancer tissue stained with mIF panels IP1 and IP2. Phenochart images of serial sections of a breast cancer tissue stained with IP1 (A) and IP2 (B) are shown. Boxes indicate ROIs selected for the analysis shown in Figure 8. Scale bars are 4 mm. (PDF 1092 KB) [file 10911_2021_9479_MOESM1_ESM.pdf]

Supplementary Figures and Tables

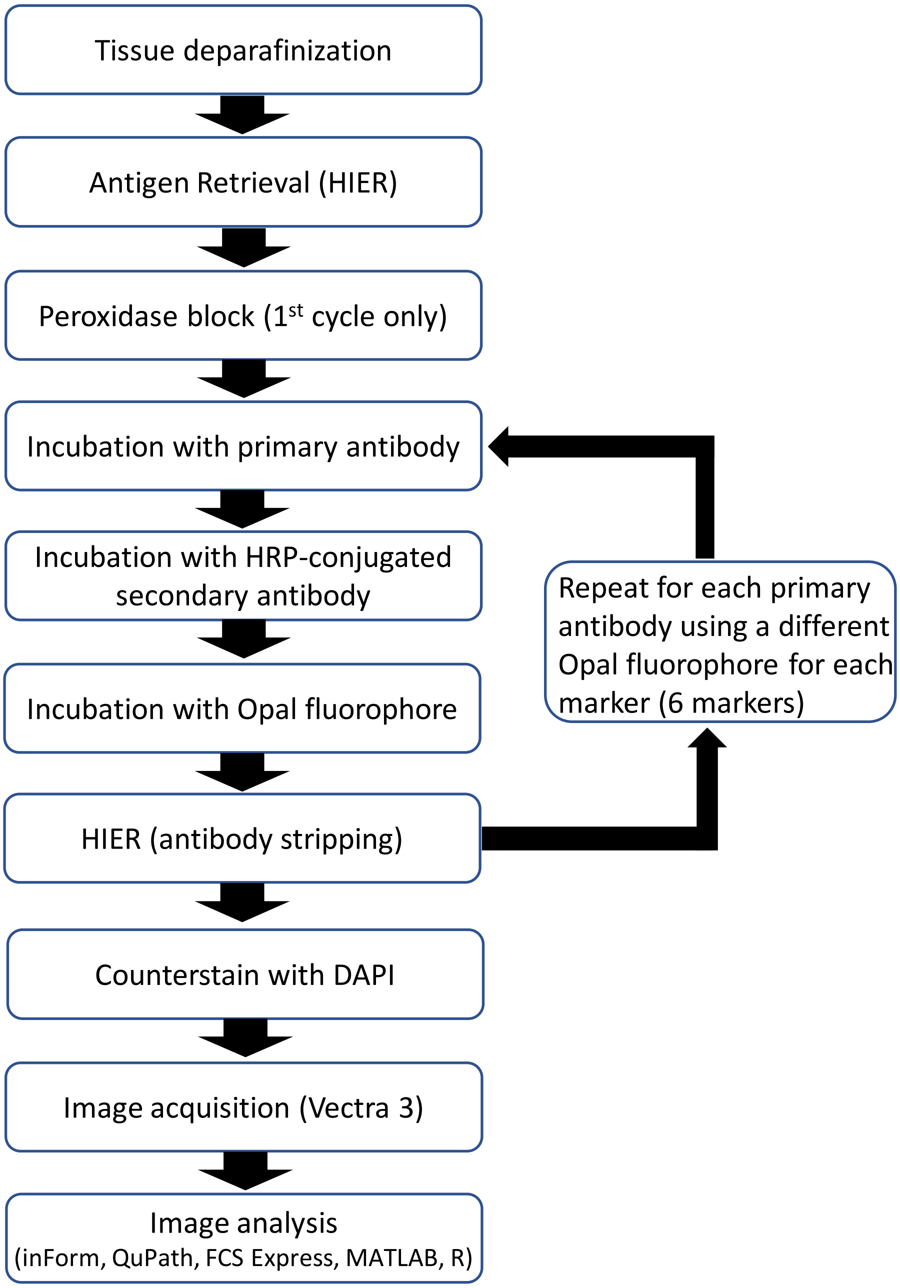

Supplementary Figure 1

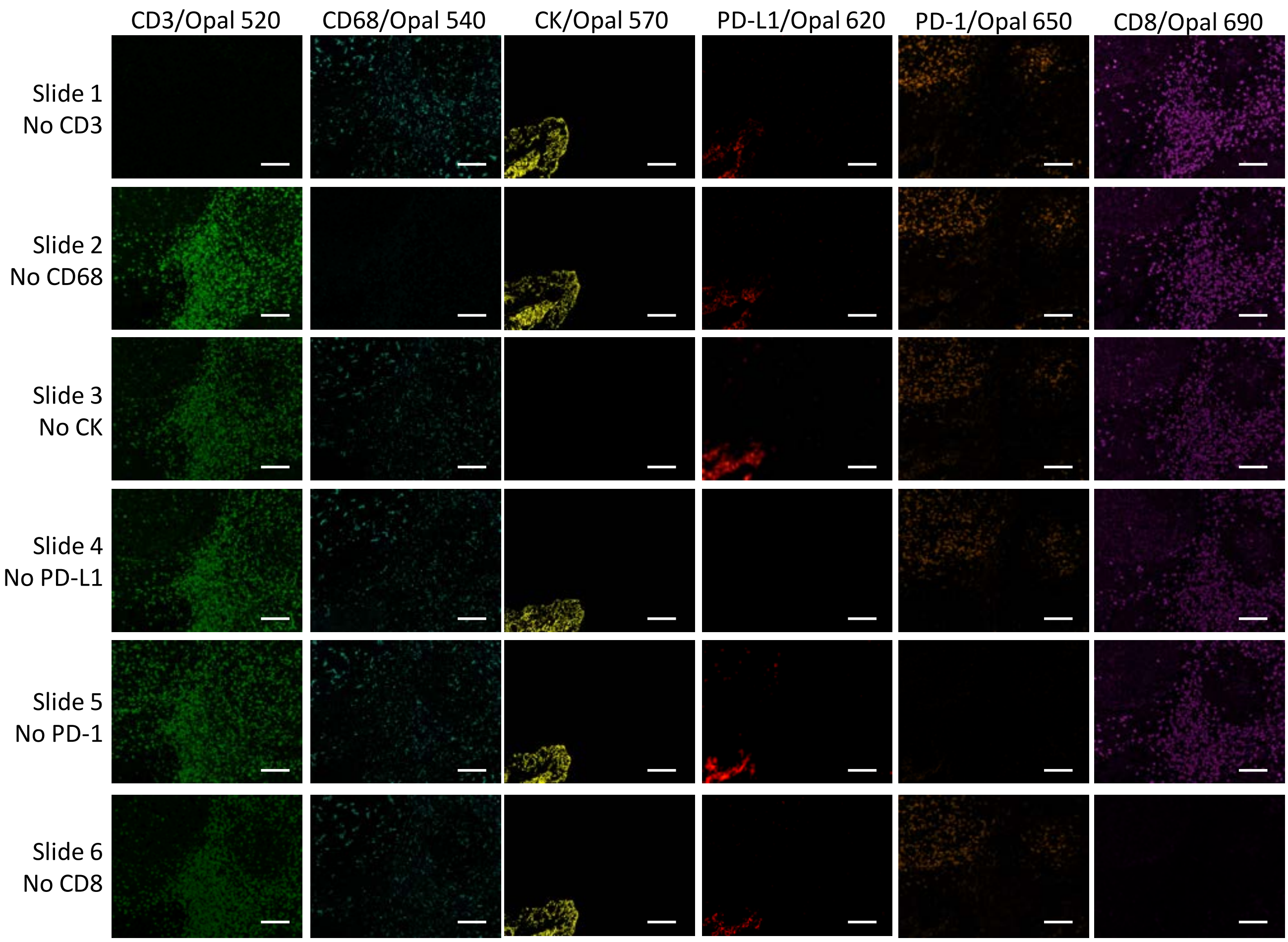

Supplementary Figure 2

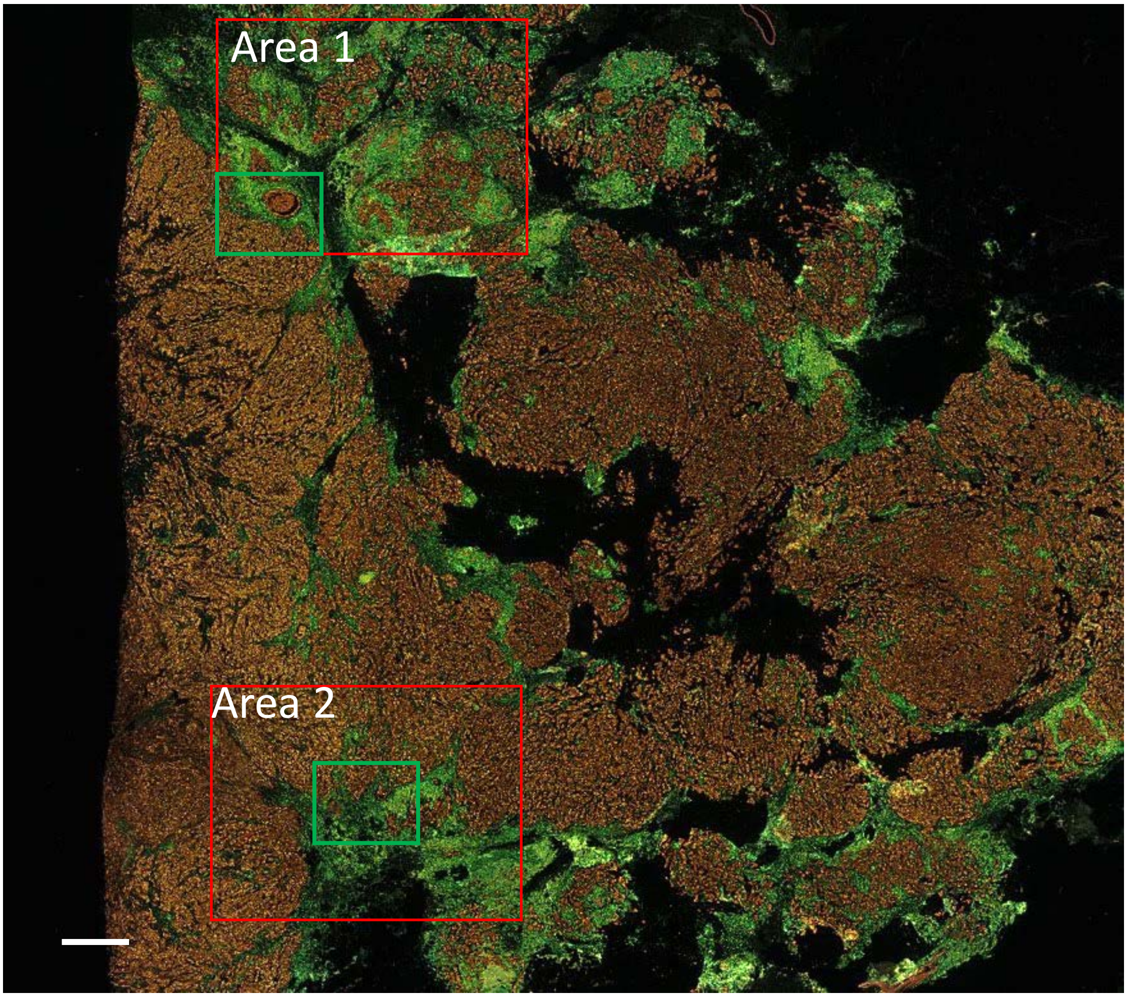

Green boxes: ROIs analyzed in Figure 5.

Supplementary Figure 3

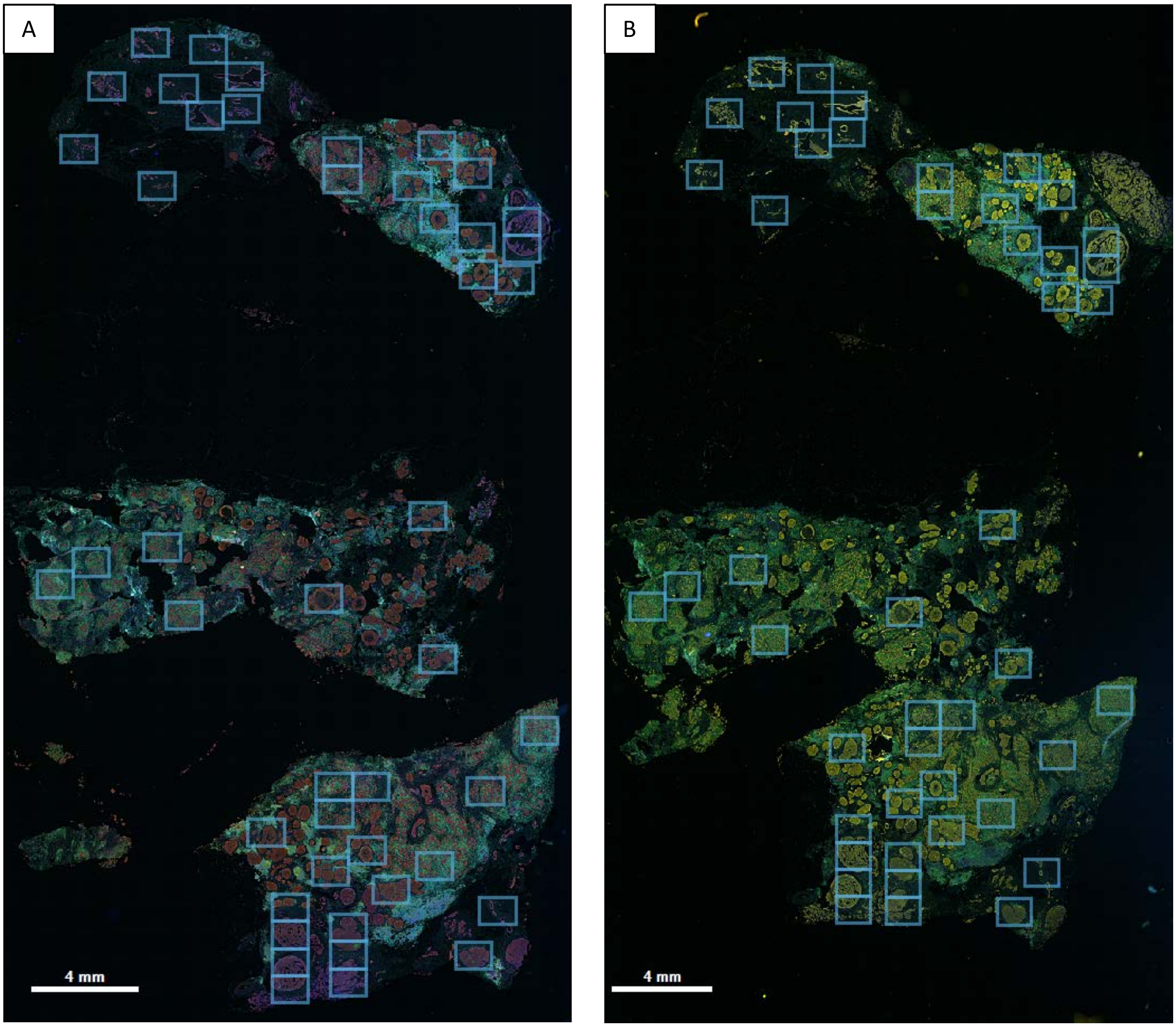

Supplementary Figure 4

Supplementary Table 1. IP1 protocol summary for Ventana Discovery.

1	First Sequence [Selected]
2	Baking [Selected]
3	Warmup Slide to [60 Deg C], and Incubate for [8 Minutes] (Baking)
4	Deparaffinization [Selected]
5	Warmup Slide to [69 Deg C], and Incubate for [8 Minutes] (Cycle 1)
6	Incubate for [8 Minutes] (Cycle 2)
7	Incubate for [8 Minutes] (Cycle 3)
8	Pretreatment [Selected]
9	Cell Conditioning [Selected]
10	CC1 Reservoir [Selected]
11	Warmup Slide to [97 Deg C], and Incubate for 4 Minutes (Cell Conditioner #1)
12	CC1 8 Min [Selected]
13	CC1 16 Min [Selected]
14	CC1 24 Min [Selected]
15	CC1 32 Min [Selected]
16	Inhibitor [Selected]
17	DISCOVERY Inhibitor [Selected]
18	Apply One Drop of DISC Inhibitor, and Incubate for [12 Minutes]
19	Antibody [Selected]
20	Apply One Drop of [PREP KIT 10] (Antibody), and Incubate for [32 Minutes]
21	Linking Antibody [Selected]
22	Multimer HRP [Selected]
23	Apply One Drop of [OMap anti-Rb HRP] (Multimer HRP), and Incubate for [24 Minutes]
24	Open Detection Kit [Selected]
25	Automated Open Detection [Selected]
26	Apply One Drop of [DETECTION 15] (Detection #23), and Incubate for [0 Hr 16 Min]
27	Dual Sequence [Selected]
28	Antibody Denaturation [Selected]
29	Antibody Denature CC2-1 [Selected]
30	Warmup Slide to [100 Deg C], and Incubate for 8 Minutes (DS CC2 denature)
31	DS Antibody [Selected]
32	Warmup Slide to [36 Deg C] from Very Low Temperatures (DS Primary Antibody)
33	Apply One Drop of [PREP KIT 14] (DS Antibody), and Incubate for [32 Minutes]
34	DS Multimer HRP [Selected]
35	Apply One Drop of [OMap anti-Ms HRP] (DS Multimer HRP), and Incubate for [24 Minutes]
36	DS Open Detection Kit [Selected]
37	DS Automated Open Detection [Selected]
38	Apply One Drop of [DETECTION 16] (Detection #24), and Incubate for [0 Hr 16 Min]
39	Triple Stain [Selected]
40	TS Antibody Denaturation [Selected]
41	Antibody Denature CC2-2 [Selected]
42	Warmup Slide to [100 Deg C], and Incubate for 8 Minutes (TS CC2 denature)
43	TS Antibody [Selected]
44	Warmup Slide to [36 Deg C] from Very Low Temperatures (TS Primary Antibody)
45	Apply One Drop of [Ki-67 (30-9)] (TS Antibody), and Incubate for [32 Minutes]
46	TS Multimer HRP [Selected]

Supplementary Table 1 (continued). IP1 protocol summary for Ventana Discovery.

47	Apply One Drop of [OMap anti-Rb HRP] (TS Multimer HRP), and Incubate for [24 Minutes]
48	TS Open Detection Kit [Selected]
49	TS Automated Open Detection [Selected]
50	Apply One Drop of [DETECTION 14] (Detection #25), and Incubate for [0 Hr 16 Min]
51	Quadruple Stain [Selected]
52	QuS Antibody Denaturation [Selected]
53	Antibody Denature CC2-3 [Selected]
54	Warmup Slide to [100 Deg C], and Incubate for 8 Minutes (QuS CC2 denature)
55	QuS Antibody [Selected]
56	Warmup Slide to [36 Deg C] from Very Low Temperatures (QuS Primary Antibody)
57	Apply One Drop of [ANTI-CD20] (QuS Antibody), and Incubate for [32 Minutes]
58	QuS Multimer HRP [Selected]
59	Apply One Drop of [OMap anti-Ms HRP] (QuS Multimer HRP), and Incubate for [24 Minutes]
60	QuS Open Detection Kit [Selected]
61	QuS Automated Open Detection [Selected]
62	Apply One Drop of [DETECTION 12] (Detection #26), and Incubate for [0 Hr 16 Min]
63	Quintuple Stain [Selected]
64	QnS Antibody Denaturation [Selected]
65	Antibody Denature CC2-4 [Selected]
66	Warmup Slide to [100 Deg C], and Incubate for 8 Minutes (QnS CC2 denature)
67	QnS Antibody [Selected]
68	Warmup Slide to [36 Deg C] from Very Low Temperatures (QnS Primary Antibody)
69	Apply One Drop of [anti-CD3 (2GV6)] (QnS Antibody), and Incubate for [32 Minutes]
70	QnS Multimer HRP [Selected]
71	Apply One Drop of [OMap anti-Rb HRP] (QnS Multimer HRP), and Incubate for [24 Minutes]
72	QnS Open Detection Kit [Selected]
73	QnS Automated Open Detection [Selected]
74	Apply One Drop of [DETECTION 1] (Detection #27), and Incubate for [0 Hr 16 Min]
75	Sextuple Stain [Selected]
76	SxS Antibody Denaturation [Selected]
77	Antibody Denature CC2-5 [Selected]
78	Warmup Slide to [100 Deg C], and Incubate for 8 Minutes (SxS CC2 Denature)
79	SxS Antibody [Selected]
80	Warmup Slide to [36 Deg C] from Very Low Temperatures (SxS Primary Antibody)
81	Apply One Drop of [PREP KIT 19] (SxS Antibody), and Incubate for [32 Minutes]
82	SxS Multimer HRP [Selected]
83	Apply One Drop of [OMap anti-Rb HRP] (SxS Multimer HRP), and Incubate for [24 Minutes]
84	SxS Open Detection Kit [Selected]
85	SxS Automated Open Detection [Selected]
86	Apply One Drop of [DETECTION 11] (Detection #28), and Incubate for [0 Hr 16 Min]
87	Counterstain [Selected]
88	Use DW for Counterstain [Selected]
89	Apply One Drop of [COUNTERSTAIN 1] (Counterstain), and Incubate for [4 Minutes]

Supplementary Table 2. IP2 protocol summary for Ventana Discovery.

1 First Sequence [Selected]
2 Baking [Selected]
3 Warmup Slide to [60 Deg C], and Incubate for [8 Minutes] (Baking)
4 Deparaffinization [Selected]
5 Warmup Slide to [69 Deg C], and Incubate for [8 Minutes] (Cycle 1)
6 Incubate for [8 Minutes] (Cycle 2)
7 Incubate for [8 Minutes] (Cycle 3)
8 Pretreatment [Selected]
9 Cell Conditioning [Selected]
10 CC1 Reservoir [Selected]
11 Warmup Slide to [97 Deg C], and Incubate for 4 Minutes (Cell Conditioner #1)
12 CC1 8 Min [Selected]
13 CC1 16 Min [Selected]
14 CC1 24 Min [Selected]
15 CC1 32 Min [Selected]
16 CC1 40 Min [Selected]
17 CC1 48 Min [Selected]
18 CC1 56 Min [Selected]
19 CC1 64 Min [Selected]
20 CC1 72 Min [Selected]
21 CC1 80 Min [Selected]
22 CC1 88 Min [Selected]
23 CC1 92 Min [Selected]
24 Inhibitor [Selected]
25 DISCOVERY Inhibitor [Selected]
26 Apply One Drop of DISC Inhibitor, and Incubate for [12 Minutes]
27 Antibody [Selected]
28 Apply One Drop of [PREP KIT 12] (Antibody), and Incubate for [60 Minutes]
29 Multimer HRP [Selected]
30 Apply One Drop of [OMap anti-Rb HRP] (Multimer HRP), and Incubate for [24 Minutes]
31 Open Detection Kit [Selected]
32 Automated Open Detection [Selected]
33 Apply One Drop of [DETECTION 15] (Detection #23), and Incubate for [0 Hr 16 Min]
34 Dual Sequence [Selected]
35 Antibody Denaturation [Selected]
36 Antibody Denature CC2-1 [Selected]
37 Warmup Slide to [100 Deg C], and Incubate for 8 Minutes (DS CC2 denature)
38 DS Antibody [Selected]
39 Warmup Slide to [36 Deg C] from Very Low Temperatures (DS Primary Antibody)
40 Apply One Drop of [PREP KIT 16] (DS Antibody), and Incubate for [32 Minutes]
41 DS Multimer HRP [Selected]
42 Apply One Drop of [OMap anti-Rb HRP] (DS Multimer HRP), and Incubate for [24 Minutes]
43 DS Open Detection Kit [Selected]
44 DS Automated Open Detection [Selected]
45 Apply One Drop of [DETECTION 16] (Detection #24), and Incubate for [0 Hr 16 Min]
46 Triple Stain [Selected]

Supplementary Table 2 (continued). IP2 protocol summary for Ventana Discovery.

47 TS Antibody Denaturation [Selected]
48 Antibody Denature CC2-2 [Selected]
49 Warmup Slide to [100 Deg C], and Incubate for 8 Minutes (TS CC2 denature)
50 TS Antibody [Selected]
51 Warmup Slide to [36 Deg C] from Very Low Temperatures (TS Primary Antibody)
52 Apply One Drop of [PREP KIT 9] (TS Antibody), and Incubate for [32 Minutes]
53 TS Multimer HRP [Selected]
54 Apply One Drop of [OMap anti-Ms HRP] (TS Multimer HRP), and Incubate for [24 Minutes]
55 TS Open Detection Kit [Selected]
56 TS Automated Open Detection [Selected]
57 Apply One Drop of [DETECTION 14] (Detection #25), and Incubate for [0 Hr 16 Min]
58 Quadruple Stain [Selected]
59 QuS Antibody Denaturation [Selected]
60 Antibody Denature CC2-3 [Selected]
61 Warmup Slide to [100 Deg C], and Incubate for 8 Minutes (QuS CC2 denature)
62 QuS Antibody [Selected]
63 Warmup Slide to [36 Deg C] from Very Low Temperatures (QuS Primary Antibody)
64 Apply One Drop of [PREP KIT 14] (QuS Antibody), and Incubate for [32 Minutes]
65 QuS Multimer HRP [Selected]
66 Apply One Drop of [OMap anti-Ms HRP] (QuS Multimer HRP), and Incubate for [24 Minutes]
67 QuS Open Detection Kit [Selected]
68 QuS Automated Open Detection [Selected]
69 Apply One Drop of [DETECTION 11] (Detection #26), and Incubate for [0 Hr 16 Min]
70 Quintuple Stain [Selected]
71 QnS Antibody Denaturation [Selected]
72 Antibody Denature CC2-4 [Selected]
73 Warmup Slide to [100 Deg C], and Incubate for 8 Minutes (QnS CC2 denature)
74 QnS Antibody [Selected]
75 Warmup Slide to [36 Deg C] from Very Low Temperatures (QnS Primary Antibody)
76 Apply One Drop of [PREP KIT 18] (QnS Antibody), and Incubate for [32 Minutes]
77 QnS Multimer HRP [Selected]
78 Apply One Drop of [OMap anti-Ms HRP] (QnS Multimer HRP), and Incubate for [24 Minutes]
79 QnS Open Detection Kit [Selected]
80 QnS Automated Open Detection [Selected]
81 Apply One Drop of [DETECTION 12] (Detection #27), and Incubate for [0 Hr 16 Min]
82 Sextuple Stain [Selected]
83 SxS Antibody Denaturation [Selected]
84 Antibody Denature CC2-5 [Selected]
85 Warmup Slide to [100 Deg C], and Incubate for 8 Minutes (SxS CC2 Denature)
86 SxS Antibody [Selected]
87 Warmup Slide to [36 Deg C] from Very Low Temperatures (SxS Primary Antibody)
88 Apply One Drop of [anti-CD3 (2GV6)] (SxS Antibody), and Incubate for [32 Minutes]
89 SxS Multimer HRP [Selected]
90 Apply One Drop of [OMap anti-Rb HRP] (SxS Multimer HRP), and Incubate for [24 Minutes]
91 SxS Open Detection Kit [Selected]
92 SxS Automated Open Detection [Selected]
93 Apply One Drop of [DETECTION 1] (Detection #28), and Incubate for [0 Hr 16 Min]
94 Counterstain [Selected]
95 Use DW for Counterstain [Selected]
96 Apply One Drop of [COUNTERSTAIN 1] (Counterstain), and Incubate for [4 Minutes]
