# Supplementary material for: Characterizing the Tumor Immune Microenvironment with Tyramide-Based Multiplex Immunofluorescence
Source: J Mammary Gland Biol Neoplasia. 2021 Feb 15;25(4):417–32. doi: 10.1007/s10911-021-09479-2 (PMC7960613; doi:10.1007/s10911-021-09479-2)
Supplement: Supplementary file 2 — Supplementary file2: Table 1. IP1 protocol summary for Ventana Discovery. Table 2. IP2 protocol summary for Ventana Discovery. (PDF 548 KB) [file 10911_2021_9479_MOESM2_ESM.pdf]

Supplementary Table 1. IP1 protocol summary for Ventana Discovery.

1	First Sequence [Selected]
2	Baking [Selected]
3	Warmup Slide to [60 Deg C], and Incubate for [8 Minutes] (Baking)
4	Deparaffinization [Selected]
5	Warmup Slide to [69 Deg C], and Incubate for [8 Minutes] (Cycle 1)
6	Incubate for [8 Minutes] (Cycle 2)
7	Incubate for [8 Minutes] (Cycle 3)
8	Pretreatment [Selected]
9	Cell Conditioning [Selected]
10	CC1 Reservoir [Selected]
11	Warmup Slide to [97 Deg C], and Incubate for 4 Minutes (Cell Conditioner #1)
12	CC1 8 Min [Selected]
13	CC1 16 Min [Selected]
14	CC1 24 Min [Selected]
15	CC1 32 Min [Selected]
16	Inhibitor [Selected]
17	DISCOVERY Inhibitor [Selected]
18	Apply One Drop of DISC Inhibitor, and Incubate for [12 Minutes]
19	Antibody [Selected]
20	Apply One Drop of [PREP KIT 10] (Antibody), and Incubate for [32 Minutes]
21	Linking Antibody [Selected]
22	Multimer HRP [Selected]
23	Apply One Drop of [OMap anti-Rb HRP] (Multimer HRP), and Incubate for [24 Minutes]
24	Open Detection Kit [Selected]
25	Automated Open Detection [Selected]
26	Apply One Drop of [DETECTION 15] (Detection #23), and Incubate for [0 Hr 16 Min]
27	Dual Sequence [Selected]
28	Antibody Denaturation [Selected]
29	Antibody Denature CC2-1 [Selected]
30	Warmup Slide to [100 Deg C], and Incubate for 8 Minutes (DS CC2 denature)
31	DS Antibody [Selected]
32	Warmup Slide to [36 Deg C] from Very Low Temperatures (DS Primary Antibody)
33	Apply One Drop of [PREP KIT 14] (DS Antibody), and Incubate for [32 Minutes]
34	DS Multimer HRP [Selected]
35	Apply One Drop of [OMap anti-Ms HRP] (DS Multimer HRP), and Incubate for [24 Minutes]
36	DS Open Detection Kit [Selected]
37	DS Automated Open Detection [Selected]
38	Apply One Drop of [DETECTION 16] (Detection #24), and Incubate for [0 Hr 16 Min]
39	Triple Stain [Selected]
40	TS Antibody Denaturation [Selected]
41	Antibody Denature CC2-2 [Selected]
42	Warmup Slide to [100 Deg C], and Incubate for 8 Minutes (TS CC2 denature)
43	TS Antibody [Selected]
44	Warmup Slide to [36 Deg C] from Very Low Temperatures (TS Primary Antibody)
45	Apply One Drop of [Ki-67 (30-9)] (TS Antibody), and Incubate for [32 Minutes]
46	TS Multimer HRP [Selected]

Supplementary Table 1 (continued). IP1 protocol summary for Ventana Discovery.

47 Apply One Drop of [OMap anti-Rb HRP] (TS Multimer HRP), and Incubate for [24 Minutes]
48 TS Open Detection Kit [Selected]
49 TS Automated Open Detection [Selected]
50 Apply One Drop of [DETECTION 14] (Detection #25), and Incubate for [0 Hr 16 Min]
51 Quadruple Stain [Selected]
52 QuS Antibody Denaturation [Selected]
53 Antibody Denature CC2-3 [Selected]
54 Warmup Slide to [100 Deg C], and Incubate for 8 Minutes (QuS CC2 denature)
55 QuS Antibody [Selected]
56 Warmup Slide to [36 Deg C] from Very Low Temperatures (QuS Primary Antibody)
57 Apply One Drop of [ANTI-CD20] (QuS Antibody), and Incubate for [32 Minutes]
58 QuS Multimer HRP [Selected]
59 Apply One Drop of [OMap anti-Ms HRP] (QuS Multimer HRP), and Incubate for [24 Minutes]
60 QuS Open Detection Kit [Selected]
61 QuS Automated Open Detection [Selected]
62 Apply One Drop of [DETECTION 12] (Detection #26), and Incubate for [0 Hr 16 Min]
63 Quintuple Stain [Selected]
64 QnS Antibody Denaturation [Selected]
65 Antibody Denature CC2-4 [Selected]
66 Warmup Slide to [100 Deg C], and Incubate for 8 Minutes (QnS CC2 denature)
67 QnS Antibody [Selected]
68 Warmup Slide to [36 Deg C] from Very Low Temperatures (QnS Primary Antibody)
69 Apply One Drop of [anti-CD3 (2GV6)] (QnS Antibody), and Incubate for [32 Minutes]
70 QnS Multimer HRP [Selected]
71 Apply One Drop of [OMap anti-Rb HRP] (QnS Multimer HRP), and Incubate for [24 Minutes]
72 QnS Open Detection Kit [Selected]
73 QnS Automated Open Detection [Selected]
74 Apply One Drop of [DETECTION 1] (Detection #27), and Incubate for [0 Hr 16 Min]
75 Sextuple Stain [Selected]
76 SxS Antibody Denaturation [Selected]
77 Antibody Denature CC2-5 [Selected]
78 Warmup Slide to [100 Deg C], and Incubate for 8 Minutes (SxS CC2 Denature)
79 SxS Antibody [Selected]
80 Warmup Slide to [36 Deg C] from Very Low Temperatures (SxS Primary Antibody)
81 Apply One Drop of [PREP KIT 19] (SxS Antibody), and Incubate for [32 Minutes]
82 SxS Multimer HRP [Selected]
83 Apply One Drop of [OMap anti-Rb HRP] (SxS Multimer HRP), and Incubate for [24 Minutes]
84 SxS Open Detection Kit [Selected]
85 SxS Automated Open Detection [Selected]
86 Apply One Drop of [DETECTION 11] (Detection #28), and Incubate for [0 Hr 16 Min]
87 Counterstain [Selected]
88 Use DW for Counterstain [Selected]
89 Apply One Drop of [COUNTERSTAIN 1] (Counterstain), and Incubate for [4 Minutes]

Supplementary Table 2. IP2 protocol summary for Ventana Discovery.

1 First Sequence [Selected]
2 Baking [Selected]
3 Warmup Slide to [60 Deg C], and Incubate for [8 Minutes] (Baking)
4 Deparaffinization [Selected]
5 Warmup Slide to [69 Deg C], and Incubate for [8 Minutes] (Cycle 1)
6 Incubate for [8 Minutes] (Cycle 2)
7 Incubate for [8 Minutes] (Cycle 3)
8 Pretreatment [Selected]
9 Cell Conditioning [Selected]
10 CC1 Reservoir [Selected]
11 Warmup Slide to [97 Deg C], and Incubate for 4 Minutes (Cell Conditioner #1)
12 CC1 8 Min [Selected]
13 CC1 16 Min [Selected]
14 CC1 24 Min [Selected]
15 CC1 32 Min [Selected]
16 CC1 40 Min [Selected]
17 CC1 48 Min [Selected]
18 CC1 56 Min [Selected]
19 CC1 64 Min [Selected]
20 CC1 72 Min [Selected]
21 CC1 80 Min [Selected]
22 CC1 88 Min [Selected]
23 CC1 92 Min [Selected]
24 Inhibitor [Selected]
25 DISCOVERY Inhibitor [Selected]
26 Apply One Drop of DISC Inhibitor, and Incubate for [12 Minutes]
27 Antibody [Selected]
28 Apply One Drop of [PREP KIT 12] (Antibody), and Incubate for [60 Minutes]
29 Multimer HRP [Selected]
30 Apply One Drop of [OMap anti-Rb HRP] (Multimer HRP), and Incubate for [24 Minutes]
31 Open Detection Kit [Selected]
32 Automated Open Detection [Selected]
33 Apply One Drop of [DETECTION 15] (Detection #23), and Incubate for [0 Hr 16 Min]
34 Dual Sequence [Selected]
35 Antibody Denaturation [Selected]
36 Antibody Denature CC2-1 [Selected]
37 Warmup Slide to [100 Deg C], and Incubate for 8 Minutes (DS CC2 denature)
38 DS Antibody [Selected]
39 Warmup Slide to [36 Deg C] from Very Low Temperatures (DS Primary Antibody)
40 Apply One Drop of [PREP KIT 16] (DS Antibody), and Incubate for [32 Minutes]
41 DS Multimer HRP [Selected]
42 Apply One Drop of [OMap anti-Rb HRP] (DS Multimer HRP), and Incubate for [24 Minutes]
43 DS Open Detection Kit [Selected]
44 DS Automated Open Detection [Selected]
45 Apply One Drop of [DETECTION 16] (Detection #24), and Incubate for [0 Hr 16 Min]
46 Triple Stain [Selected]

Supplementary Table 2 (continued). IP2 protocol summary for Ventana Discovery.

47	TS Antibody Denaturation [Selected]
48	Antibody Denature CC2-2 [Selected]
49	Warmup Slide to [100 Deg C], and Incubate for 8 Minutes (TS CC2 denature)
50	TS Antibody [Selected]
51	Warmup Slide to [36 Deg C] from Very Low Temperatures (TS Primary Antibody)
52	Apply One Drop of [PREP KIT 9] (TS Antibody), and Incubate for [32 Minutes]
53	TS Multimer HRP [Selected]
54	Apply One Drop of [OMap anti-Ms HRP] (TS Multimer HRP), and Incubate for [24 Minutes]
55	TS Open Detection Kit [Selected]
56	TS Automated Open Detection [Selected]
57	Apply One Drop of [DETECTION 14] (Detection #25), and Incubate for [0 Hr 16 Min]
58	Quadruple Stain [Selected]
59	QuS Antibody Denaturation [Selected]
60	Antibody Denature CC2-3 [Selected]
61	Warmup Slide to [100 Deg C], and Incubate for 8 Minutes (QuS CC2 denature)
62	QuS Antibody [Selected]
63	Warmup Slide to [36 Deg C] from Very Low Temperatures (QuS Primary Antibody)
64	Apply One Drop of [PREP KIT 14] (QuS Antibody), and Incubate for [32 Minutes]
65	QuS Multimer HRP [Selected]
66	Apply One Drop of [OMap anti-Ms HRP] (QuS Multimer HRP), and Incubate for [24 Minutes]
67	QuS Open Detection Kit [Selected]
68	QuS Automated Open Detection [Selected]
69	Apply One Drop of [DETECTION 11] (Detection #26), and Incubate for [0 Hr 16 Min]
70	Quintuple Stain [Selected]
71	QnS Antibody Denaturation [Selected]
72	Antibody Denature CC2-4 [Selected]
73	Warmup Slide to [100 Deg C], and Incubate for 8 Minutes (QnS CC2 denature)
74	QnS Antibody [Selected]
75	Warmup Slide to [36 Deg C] from Very Low Temperatures (QnS Primary Antibody)
76	Apply One Drop of [PREP KIT 18] (QnS Antibody), and Incubate for [32 Minutes]
77	QnS Multimer HRP [Selected]
78	Apply One Drop of [OMap anti-Ms HRP] (QnS Multimer HRP), and Incubate for [24 Minutes]
79	QnS Open Detection Kit [Selected]
80	QnS Automated Open Detection [Selected]
81	Apply One Drop of [DETECTION 12] (Detection #27), and Incubate for [0 Hr 16 Min]
82	Sextuple Stain [Selected]
83	SxS Antibody Denaturation [Selected]
84	Antibody Denature CC2-5 [Selected]
85	Warmup Slide to [100 Deg C], and Incubate for 8 Minutes (SxS CC2 Denature)
86	SxS Antibody [Selected]
87	Warmup Slide to [36 Deg C] from Very Low Temperatures (SxS Primary Antibody)
88	Apply One Drop of [anti-CD3 (2GV6)] (SxS Antibody), and Incubate for [32 Minutes]
89	SxS Multimer HRP [Selected]
90	Apply One Drop of [OMap anti-Rb HRP] (SxS Multimer HRP), and Incubate for [24 Minutes]
91	SxS Open Detection Kit [Selected]
92	SxS Automated Open Detection [Selected]
93	Apply One Drop of [DETECTION 1] (Detection #28), and Incubate for [0 Hr 16 Min]
94	Counterstain [Selected]
95	Use DW for Counterstain [Selected]
96	Apply One Drop of [COUNTERSTAIN 1] (Counterstain), and Incubate for [4 Minutes]
